# Evodiamine Exerts an Anti-Hepatocellular Carcinoma Activity through a WWOX-Dependent Pathway

**DOI:** 10.3390/molecules22071175

**Published:** 2017-07-14

**Authors:** Che-Yuan Hu, Hung-Tsung Wu, Yu-Chu Su, Ching-Han Lin, Chih-Jen Chang, Chao-Liang Wu

**Affiliations:** 1 Graduate Institute of Clinical Medicine, College of Medicine, National Cheng Kung University, Tainan 701, Taiwan; greatoldhu@gmail.com (C.-Y.H.); cyclops0113@yahoo.com.tw (C.-H.L.); 2 Department of Urology, National Cheng Kung University Hospital, College of Medicine, National Cheng Kung University, Tainan 704, Taiwan; 3 Research Center of Clinical Medicine, National Cheng Kung University Hospital, College of Medicine, National Cheng Kung University, Tainan 704, Taiwan; microbe0905702@yahoo.com.tw (H.-T.W.); horseofblackchu@gmail.com (Y.-C.S.); 4 Department of Family Medicine, National Cheng Kung University Hospital, College of Medicine, National Cheng Kung University, Tainan 704, Taiwan; 5 Department of Otolaryngology, National Cheng Kung University Hospital, College of Medicine, National Cheng Kung University, Tainan 704, Taiwan; 6 Department of Biochemistry and Molecular Biology, College of Medicine, National Cheng Kung University, Tainan 701, Taiwan; 7 Division of Endocrinology and Metabolism, Department of Internal Medicine, National Cheng Kung University Hospital, College of Medicine, National Cheng Kung University, Tainan 704, Taiwan

**Keywords:** *Evodia rutaecarpa*, evodiamine, hepatocellular carcinoma, herbal medicine, WW domain-containing oxidoreductase

## Abstract

Evodiamine is one of the main components isolated from *Evodia rutaecarpa*, and it has been reported to exert inhibitory effects on cancers by anti-proliferative and apoptosis-inducing activities. Although the anti-cancer activity of evodiamine has been identified, the precise mechanisms of this action remain obscure. While previous studies indicated that evodiamine exerts anti-tumor effects through inhibiting β-catenin activity, and WW domain-containing oxidoreductase (WWOX) regulates β-catenin accumulation in cytoplasm, the effects of evodiamine on the expression of WWOX are still unknown. In this study, we provide evidence that evodiamine dose- and time-dependently inhibits both *Mus musculus* and *Homo sapiens* hepatocellular carcinoma (HCC) cells, as well as Hepa1-6 and HepG2 cell proliferation. We further tested the therapeutic effects of evodiamine in Hepa1-6 hepatoma-bearing mice, and we found that treatment of evodiamine by oral gavage significantly decreased the tumor size of the mice. Moreover, the expressions of WWOX were dose-dependently increased in HCC cell lines as well as in Hepa1-6 hepatoma-bearing mice after the treatment with evodiamine. Knockdown of WWOX in HepG2 and Hepa1-6 cells diminished the effects of evodiamine on the inhibitory effect of cancer cell growth, indicating that evodiamine induced anti-cancer activity through a WWOX-dependent pathway. As such, evodiamine activated WWOX to exert an anti-HCC activity, and might be a potential therapeutic or preventive candidate for HCC treatment.

## 1. Introduction

Hepatocellular carcinoma (HCC) is the most predominant type of primary liver cancer, and the second leading cause of cancer-related mortality worldwide [1]. Radiofrequency ablation, surgical resection, and liver transplantation are the primary curative treatments for HCC. In addition, Sorafenib is the only agent proven to significantly prolong a patient’s survival [2]. However, the costs of these treatments are still very high, and they are not available in many countries. Therefore, effective treatment for advanced HCC remains a major challenge, and new agents are needed for advanced HCC or adjuvant treatment after resection or ablation in patients with early HCC.

WW domain-containing oxidoreductase (WWOX) is a tumor suppressor gene, and reduced expression of WWOX is noted in multiple cancers such as HCC, non-small cell lung cancer, osteosarcoma, breast cancer, and prostate cancer [3]. WWOX has been reported to be downregulated in HCC cell lines as well as in primary HCC tissues, and WWOX is implicated in the Wnt/β-catenin pathway, which is frequently affected in HCC [4,5,6]. In HCC, the downregulation of WWOX is associated with β-catenin accumulation in cytoplasm, and β-catenin will subsequently translocate into the nucleus and activate Wnt/β-catenin target genes [4]. The activation of the Wnt/β-catenin signaling pathway will enhance the proliferation and cell-cycle progression of HCC [7]. The activation of WWOX is thus a novel strategy for the treatment of HCC.

Evodiamine, a kind of quinazolinocarboline alkaloid, is one of the components isolated from a Chinese herbal medicine, called Wu-Chu-Yu (*Evodia rutaecarpa*). Evodiamine has been shown to improve cognitive abilities, have anti-inflammatory activity, and be able to address circulation failure. In addition, it is hypothesized to have vasorelaxant and cardiotonic effects [8]. Recently, some studies revealed that evodiamine has an anti-tumor effect on the gastrointestinal [9] and genitourinary tracts [10]. Several studies have demonstrated that evodiamine induces cell cycle arrest at the G2/M phase and further activates apoptosis, although the precise mechanisms for this action remain obscure [10]. While evodiamine exerts anti-tumor effects through inhibiting β-catenin activity [11], and WWOX regulates β-catenin accumulation in cytoplasm, the effects of evodiamine on the expression of WWOX are also still unknown.

In the present study, we thus aimed to investigate the possible mechanisms of evodiamine in the anti-tumor activity with regard to HCC in both in vitro and in vivo models. In addition, the related role of WWOX in the anti-HCC activity of evodiamine was also clarified.

## 2. Material and Methods

### 2.1. Cell Cultures

The hepatoma cell lines, HepG2 and Hepa1-6, were purchased from the Bioresource Collection and Research Center (Food Industry Research and Development Institute, Hsinchu, Taiwan). Cells were maintained in Dulbecco’s modified Eagle medium (Hyclone, South Logan, UT, USA) supplemented with 10% heat-inactivated fetal bovine serum.

### 2.2. MTT Assay

Cells (5 × 10^4^ cells per well) were seeded in a 96-well flat-bottom culture plate. After additional treatment for the indicated intervals, 100 μL of 0.2 mg/mL 3-(4,5-methylthiazol-2-yl)-2,5-diphenyl-tetrazolium bromide (MTT, USB Corporation, Cleveland, OH, USA) was added to each well, and the cells were incubated for three hours at 37 °C. After incubation, the MTT reagent was discarded, and 100 μL of dimethyl sulfoxide was then added. The experiment was performed at room temperature for twenty minutes. The absorbance was then measured with a Multiskan GO spectrophotometer (Thermo Scientific, Waltham, MA, USA) at a wavelength of 570 nm.

### 2.3. Small Interfering Ribonucleic Acid Transfection

Cells were transfected with duplexed RNA oligonucleotides of human or mouse WWOX (Santa Cruz Biotechnology Inc., Santa Cruz, CA, USA) or scramble siRNA (as negative control) using Lipofectamine 2000 (Invitrogen, Carlsbad, CA, USA.), according to the manufacturer’s instructions. The experiments were performed at 48 h post-transfection.

### 2.4. Western Blots

The cells were harvested at the indicated times and lysed with a buffer containing 1% Triton X-100, 50 mM of Tris (pH 7.5), 10 mM of Ethylenediaminetetraacetic acid (EDTA), 0.02% NaN_3_, and a protease inhibitor cocktail (Sigma-Aldrich, St. Louis, MO, USA). The protein concentration was determined using a bicinchoninic acid (BCA) assay kit (Pierce Biotechnology, Rockford, IL, USA). Protein lysates (30 μg) were separated using 10% sodium dodecyl sulphate (SDS)-polyacrylamide gel electrophoresis and transferred to a polyvinylidene difluoride membrane (Millipore, Billerica, MA, USA). The membrane was blocked at room temperature for 1 h in tris-buffered saline (TBS)-T (10 mM Tris, 150 mM NaCl, and 0.05% Tween 20, (pH 7.6)), containing 10% skim milk, and probed with 1:1000 primary antibodies, such as WWOX (Proteintech, Chicago, IL, USA) and actin (Santa Cruz Biotechnology Inc.) at 4 °C overnight. Subsequently, the blots were washed with TBS-T and incubated with a 1:5000 dilution of horseradish peroxidase-conjugated secondary antibodies at room temperature for 1 h. The protein bands were visualized using Immobilon™ (Millipore). Actin was used as the internal control. The relative signal intensity was quantified using ImageJ software from W. Rasband (National Institutes of Health, Bethesda, MD, USA).

### 2.5. Hepa1-6 Hepatoma-Bearing Animal Model

Eight-week-old C57BL/6 male mice were purchased from the Animal Center of National Cheng Kung University Medical College. Mice were housed in a temperature (25 ± 1 °C) and humidity (60 ± 5%) controlled room and kept on a 12:12 light-dark cycle (light on at 0600). The animal procedures were performed according to the Guide for the Care and Use of Laboratory Animals of the National Institutes of Health, as well as the guidelines of the Animal Welfare Act. The mice were fed with a chow diet supplied with 10 or 20 mg/kg diet evodiamine (Sigma-Aldrich) for three days, and then Hepa1-6 tumor cells (10^7^ cells/100 μL) were implanted subcutaneously into the left hind groin of the experimental mice, as in a previous study [12].

### 2.6. Statistical Analysis

Data are expressed as means ± S.E.M. Student’s *t*-test was used to determine the source of significant differences where appropriate. Significance was indicated when the *p* value was less than 0.05.

## 3. Results

### 3.1. Evodiamine Dose- and Time-Dependently Inhibited Mus Musculus Hepatoma, Hepa1-6 Cell Proliferation

**Figure 1:**
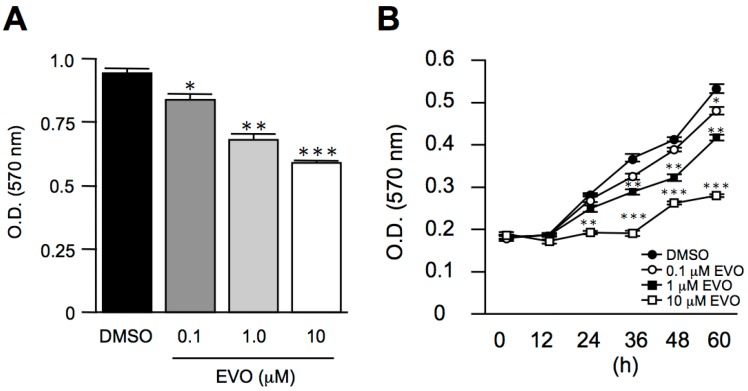
Evodiamine inhibited *Mus musculus* hepatoma, Hepa1-6 cell proliferation. *Mus musculus* hepatoma cell line and Hepa1-6 cells were treated with different doses of evodiamine for 24 h (**A**) or treated with 10 μM evodiamine and harvested at the indicated periods (**B**). The cells were then incubated with 0.2 mg/mL MTT in culture medium for three hours. The absorbance was measured at a wavelength of 570 nm. The data are expressed as means ± S.E.M. and obtained from two independent experiments, with three replicates for each experiment. * *p* < 0.05, ** *p* < 0.01, and *** *p* < 0.001 compared with the control group.

After the evodiamine treatment at a dose of 0.1 μM, we found a slight decrease in cell proliferation in Hepa1-6 cells. Following an increase in the evodiamine concentrations, the anti-proliferation activity of evodiamine gradually and significantly increased (Figure 1A). Although we found that evodiamine decreased the proliferation of Hepa1-6 cells, the mechanism by which evodiamine induced cell toxicity or cell arrest was still obscure. In order to clarify this issue, we tested the anti-proliferation activity of evodiamine in different treatment durations. As shown in Figure 1B, evodiamine arrested the cancer cell proliferation in a dose- and time-dependent manner.

### 3.2. Evodiamine Dose- and Time-Dependently Inhibited Homo Sapiens Hepatocellular Carcinoma, HepG2 Cell Proliferation

**Figure 2:**
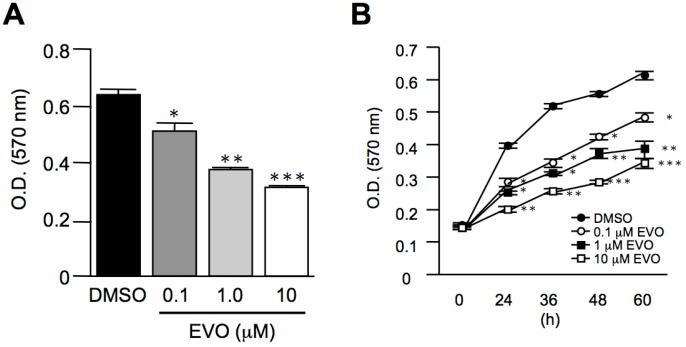
Evodiamine inhibited *Homo sapiens* hepatocellular carcinoma, HepG2 cell proliferation. *Homo sapiens* hepatocellular carcinoma and HepG2 cells were treated with different doses of evodiamine for 24 h (**A**) or treated with 10 μM evodiamine and harvested at the indicated periods (**B**). The cells were then incubated with 0.2 mg/mL MTT in culture medium for three hours. The absorbance was measured at a wavelength of 570 nm. The data are expressed as means ± S.E.M. and obtained from two independent experiments with three replicates for each experiment. * *p* < 0.05, ** *p* < 0.01, and *** *p* < 0.001 compared with the control group.

Although we found that evodiamine has an activity to inhibit *Mus musculus* hepatoma, Hepa1-6 cell proliferation (Figure 1), the effects of evodiamine in human-derived hepatoma cells are unknown. We thus investigated the effects of evodiamine in *Homo sapiens* hepatocellular carcinoma and HepG2 cell proliferation. As shown in Figure 2A, evodiamine dose-dependently inhibited HepG2 cell growth, consistent with the results found with Hepa1-6 cells. In addition, evodiamine arrested human hepatoma cell proliferation in a dose- and time-dependent manner (Figure 2B).

### 3.3. Treatment of Evodiamine Decreased Tumor Size in Hepa1-6 Hepatoma-Bearing Mice

**Figure 3:**
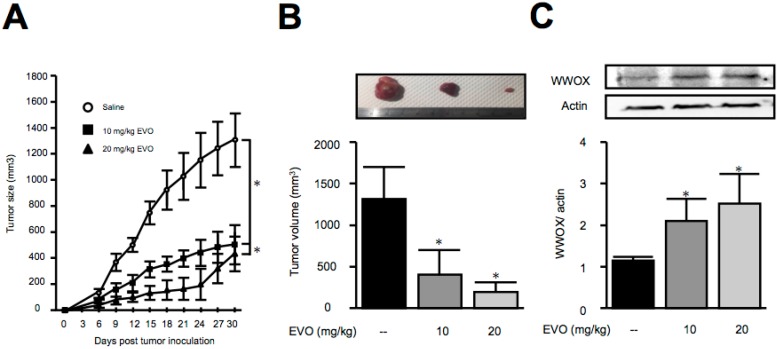
Evodiamine decreased tumor size in Hepa1-6 hepatoma-bearing mice. Hepa1-6 cells were subcutaneously injected in mice on day 0. The indicated doses of evodiamine were treated by oral gavage for 27 consecutive days. The tumor volume was determined by direct measurement with calipers and calculated every three days. (**A**) On day 30, the animals were sacrificed, and the tumors were removed (**B**) for the detection of WWOX expression (**C**). The results are expressed as means ± S.E.M. *n* = 20. * *p* < 0.05 compared with control group.

Although the anti-cancer cell proliferation activities of evodiamine in both *Mus musculus* and *Homo sapiens* were identified, the anti-tumor activity of evodiamine in vivo is unknown. In view of this issue, we established a Hepa1-6 hepatoma-bearing mice model to investigate the effects of evodiamine in animals. As shown in Figure 3, evodiamine treatment by oral gavage with different doses significantly decreased the tumor size in Hepa1-6 hepatoma-bearing mice, implying the therapeutic potential of evodiamine for the treatment of hepatocellular carcinoma.

### 3.4. Evodiamine Dose-Dependently Increased the Expression of WWOX in Both HepG2 and Hepa1-6 Hepatoma Cells

**Figure 4:**
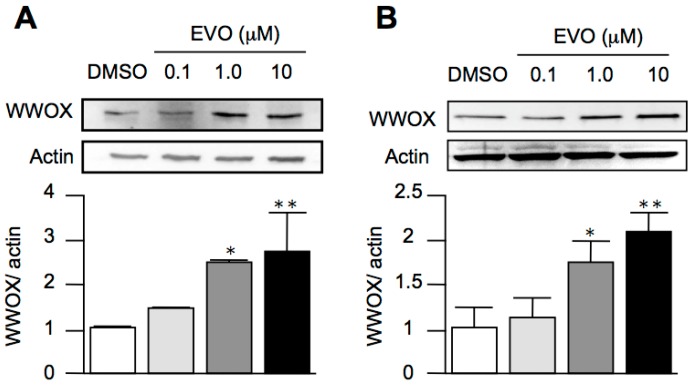
Evodiamine increased the expression of WWOX in both liver cancer cell lines. *Mus musculus* (**A**) and *Homo sapiens* (**B**) hepatocellular carcinoma cells were treated with different doses of evodiamine, as indicated, for 12 h. The WWOX levels were determined by Western blot analysis. The data are expressed as means ± S.E.M. and obtained from four independent experiments. * *p* < 0.05, and ** *p* < 0.01 compared with the control group.

Although we established the anti-tumor effects of evodiamine in vitro and in vivo, the precise mechanisms of these effects are unknown. In our results, we found that evodiamine induced cell arrest, and it is known that the activation of WWOX induces cell arrest to exert an anti-tumor activity. However, the effects of evodiamine on WWOX expression are unknown. As shown in Figure 4, evodiamine treatment dose-dependently increased the expression of WWOX in both *Mus musculus* (Figure 4A) and *Homo sapiens* (Figure 4B) hepatocellular carcinoma cells, implying that evodiamine might activate WWOX to exert an anti-tumor activity.

### 3.5. Evodiamine Exerted Anti-Cancer Activity through a WWOX-Dependent Pathway 

**Figure 5:**
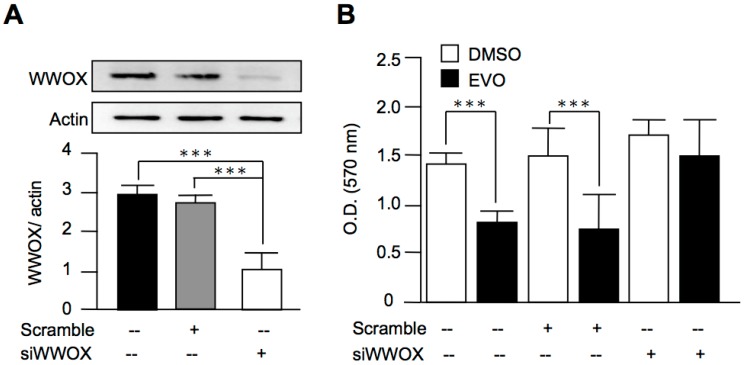
Evodiamine inhibited HepG2 cell proliferation through a WWOX-dependent pathway. (**A**) HepG2 cells were treated with scramble siRNA or siRNA targeted to WWOX (siWWOX). The knockdown of WWOX was evaluated using Western blot analysis; (**B**) After the transfection of scramble siRNA or siWWOX for 48 h, the cells were treated with 10 μM evodiamine for 24 h. The cell viability was determined by MTT assay. The data are expressed as means ± S.E.M. and obtained from four independent experiments. *** *p* < 0.001 as compared with the indicated groups.

**Figure 6:**
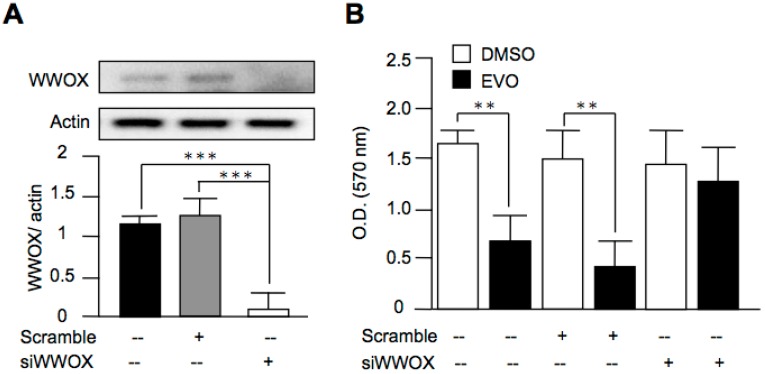
Evodiamine inhibited Hepa1-6 cell proliferation through a WWOX-dependent pathway. (**A**) Hepa1-6 cells were treated with scramble siRNA or siRNA targeted to WWOX (siWWOX). The knockdown of WWOX was evaluated using Western blot analysis; (**B**) After the transfection of scramble siRNA or siWWOX for 48 h, the cells were treated with 10 μM evodiamine for 24 h. The cell viability was determined by MTT assay. The data are expressed as means ± S.E.M. and obtained from two independent experiments. ** *p* < 0.01; *** *p* < 0.001 as compared with the indicated groups.

Although we found that evodiamine increased the expression of WWOX, the role of WWOX in the anti-cancer activity of evodiamine was still obscure. In order to clarify this issue, we downregulated the expression of WWOX in both HepG2 cells (Figure 5A) and Hepa1-6 cells (Figure 6A) using siRNAs. After the treatment of evodiamine, the inhibition activity of cell growth by evodiamine was diminished in the WWOX-knockdown group, as compared with the scramble group (Figure 5B and Figure 6B). These results indicated that evodiamine exerted the inhibition activity of liver cancer cell growth through a WWOX-dependent pathway. 

## 4. Discussion

In the present study, we found that evodiamine, the main component of *Evodia rutaecarpa*, exerted an inhibitory effect on hepatocellular carcinoma in vivo and in vitro, and this activity was related to the increase of WWOX expression. Many of the previous studies provided evidence using flow cytometry that evodiamine induced cancer cell apoptosis and cell arrest [13,14,15]. In the present study, we found the same results by MTT assays to be consistent with previous studies, indicating that evodiamine inhibited cancer cell growth. In addition, we elucidated a novel mechanism by which evodiamine activates WWOX to exert an anti-cancer activity. To the best of our knowledge, our study is the first report that provides evidence for this anti-tumor activity using a wild type mouse model instead of immune-deficient animals [16].

Numerous studies have demonstrated that evodiamine has an inhibitory effect on the proliferation of lung cancer [17], colorectal cancer [18], gastric cancer [19], and ovarian cancer [20]. Consistent with previous studies, we found that evodiamine inhibited HCC cell proliferation [12,15]. In addition, the mechanisms for the inhibitory effects of evodiamine on cancers were investigated, with the results showing that evodiamine has anti-proliferative and apoptosis-inducing activities. Evodiamine upregulates cyclin B1, p27, and p21 to inactivate cdc2 and pRb. These effects further induced G2/M phase cell cycle arrest in anti-cancer cell proliferation [15]. In addition, evodiamine increases the activity of caspase-3, -8, and -9 to induce cancer cell apoptosis [21]. Although the mechanisms of the anti-cancer activity of evodiamine have been well-studied, the precise mechanisms still remain unclear.

It is known that the downregulation of WWOX leads to cytoplasmic β-catenin accumulation and the subsequent activation of the Wnt/β-catenin signaling pathway in HCC [4], while decreases in the activities of Akt and GSK-3β inhibit the expression of β-catenin. Recent studies have also demonstrated that evodiamine inhibits the Wnt/β-catenin signaling pathway to inhibit the proliferation and stem cell properties of cancer cells [22], and it also decreases Akt phosphorylation to exert an anti-HCC effect [12]. These studies indicate that WWOX might play a role in the anti-cancer activity of evodiamine. In the present work, we found that evodiamine increased the expression of WWOX in both *Mus musculus* and *Homo sapiens* liver cancer cell lines, as well as in the tumor in Hepa1-6 hepatoma-bearing mice.

Taken together, we found that evodiamine is a novel WWOX inducer, and it activated WWOX to exert an anti-HCC activity. These results imply that evodiamine is a potential therapeutic or preventive candidate for HCC treatment.

## 5. Conclusions

In this study, we found that evodiamine is a novel WWOX inducer, and it activated WWOX to exert an anti-HCC activity. These results imply that evodiamine is a potential therapeutic or preventive candidate for HCC treatment.
